# A systematic evaluation of the biocompatibility of cucurbit[7]uril in mice

**DOI:** 10.1038/s41598-018-27206-6

**Published:** 2018-06-11

**Authors:** Xiangjun Zhang, Xiaoqiu Xu, Shengke Li, Lian-Hui Wang, Jianxiang Zhang, Ruibing Wang

**Affiliations:** 1 State Key Laboratory of Quality Research in Chinese Medicine, and Institute of Chinese Medical Sciences, University of Macau, Taipa, Macau China; 2 Department of Pharmaceutics, College of Pharmacy, Third Military Medical University, Chongqing, 400038 China; 3 Institute of Materia Medica, College of Pharmacy, Third Military Medical University, Chongqing, 400038 China; 4 Key Laboratory for Organic Electronics and Information Displays, Institute of Advanced Materials, Nanjing University of Posts & Telecommunications, Nanjing, 210046 China

## Abstract

As one of the most water-soluble members in the macrocyclic cucurbit[*n*]uril (CB[*n*]) family, CB[7] has attracted increasing attention in pharmaceutical and biomedical fields. Despite extensive studies regarding the potential use of CB[7] for biomedical applications, its full safety and toxicity profile in a clinically relevant model is still lacking. Herein we report the full biocompatibility profile of CB[7], administered orally, peritoneally or intravenously in mice, respectively. Body-weight changes showed no significant differences among various groups of mice after they were administered with CB[7] at a single dose of 5 g/kg orally, 500 mg/kg peritoneally and 150 mg/kg intravenously, respectively. Hematology tests, as well as hepatic and renal function biochemical markers tests, of the blood collected from these mice sacrificed 21 days after CB[7] administration all exhibited normal ranges of values that were comparable with those of the control group. Moreover, histopathological analysis on the sections of major organs (including the heart, liver, spleen, lungs and kidneys) and gastrointestinal tissues revealed no detectable injuries and inflammatory cells infiltration. Taken together, these results suggest an excellent biocompatibility profile of CB[7] in mice, which provide important foundations for further investigations and even clinical applications of CB[7] in biomedical areas.

## Introduction

**Figure 1:**
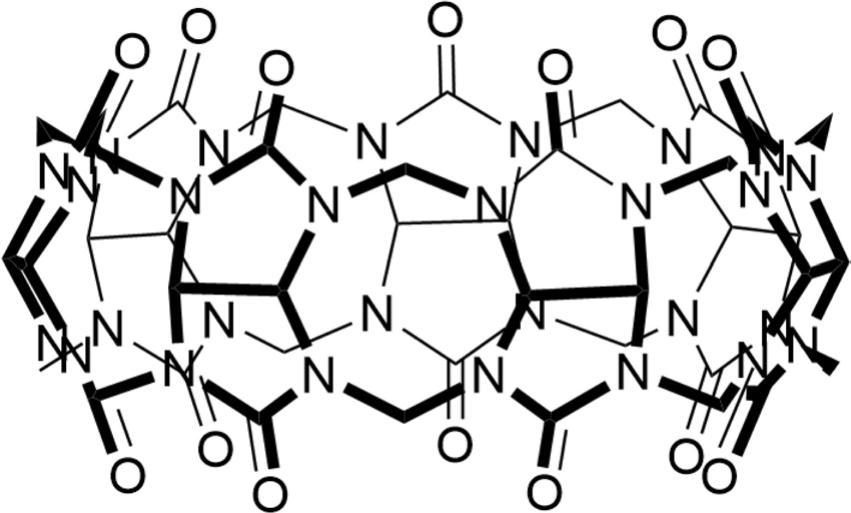
Structure of cucurbit[7]uril (CB[7]).

Due to their abilities to complex with molecules of biological interest, a variety of macrocyclic host molecules have shown great potentials in biomedical applications during the recent decades^1^. Among them, cyclodextrins (CDs) and their derivatives have been successfully developed into drug carriers or antidotes with biomedical and clinical applications^2–4^. For instance, Sugammadex, a CD derivative that selectively binds with NMBAs (neuromuscular blocking agents) such as rocuronium, has been approved as a specific antidote for the reversal of NMBAs used during various surgeries^5,6^. The success of CDs is partly attributed to their excellent biocompatibility that has been well investigated and understood^7,8^. Recently, reminiscent of CDs in many ways, cucurbit[*n*]urils (CB[*n*]s, *n* = 5–8, 10, 13–15)^9–13^, a family of macrocyclic oligomers of methylene-bridged glycolurils with the shape resembling that of a pumpkin, have emerged in pharmaceutical and biomedical sciences as synthetic receptors^14–17^. Due to its appropriate size to accommodate a variety of guest molecules of biomedical interest and its superior water solubility, CB[7] (Fig. 1) has been extensively investigated as a potential carrier for drug molecules as the molecular encapsulation by CB[7] often improved the chemical stability and solubility of the included drug molecules, controlled their release, and modulated their toxicity and therapeutic efficacies^18–26^.

To eventually leverage these unique properties that CB[7] has offered for potential biomedical applications, CB[7] must be toxicologically safe. The biocompatibility of CB[7] was previously studied with *in vitro* cell lines and demonstrated little cytotoxicity at concentrations up to the 1 mM level^27^. By using *ex vivo* models, tissue-specific toxicity investigations suggested that CB[7] exhibited measurable neurotoxicity at a 1 mM concentration level or above, various degrees of myotoxicities and cardiotoxicity at 300 μM concentration level^28^. Through the use of zebrafish models, the developmental and organ-specific toxicity of CB[7] was investigated *in vivo* and no evidence of developmental toxicity was observed at concentrations of up to 750 μM, although CB[7] was found to have measurable cardiotoxicity, as well as locomotion and behavioral toxicity at 500 μM or higher^29^. More importantly, the *in vivo* toxicity of CB[7] on a mouse model upon intravenous (*i.v*.) administration was previously studied based on body-weight changes only, and the mice exhibited reasonable tolerance towards a 250 mg/kg dosage via a slow *i.v*. injection from a body-weight change perspective^30^. In the same study, oral administration of a 1:1 CB[7]/CB[8] mixture, at a dose of 300 mg/kg:300 mg/kg, into mice did not result in any weight loss^30^. In spite of these previous efforts, there still lacks of systematic examination (beyond just body-weight changes) of the safety profile of CB[7] in a suitable preclinical model (such as mouse model) when it is administered orally (*i.g*.) or through *i.v*., or peritoneally (*i.p*.). Herein, we report our recent systematic evaluation of CB[7]’s biocompatibility profile in mice with *i.g*., *i.p*. and *i.v*. administrations, respectively, by not only monitoring the body-weight changes of the mice, but also examining their organ indices, haematological parameters, hepatic and renal function biochemical markers, as well as the histopathology of the major organ tissues of the mice.

## Results

### Toxicity evaluation of CB[7] with *i.g*. administration

**Figure 2:**
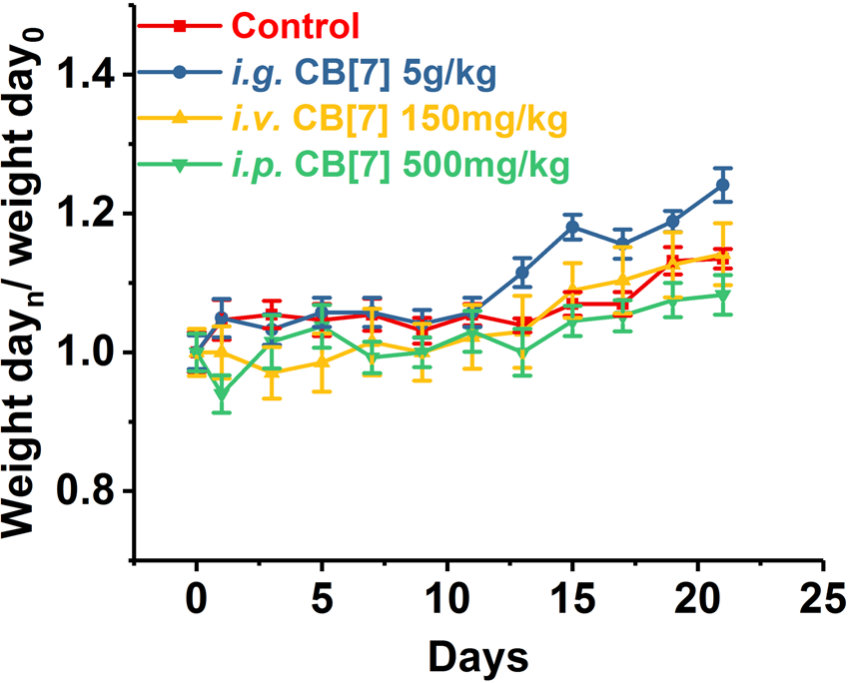
Body weight changes of the mice *i.g., i.v*. or *i.p*. administered with CB[7], respectively, monitored for 21 days post-administration.

**Figure 3:**
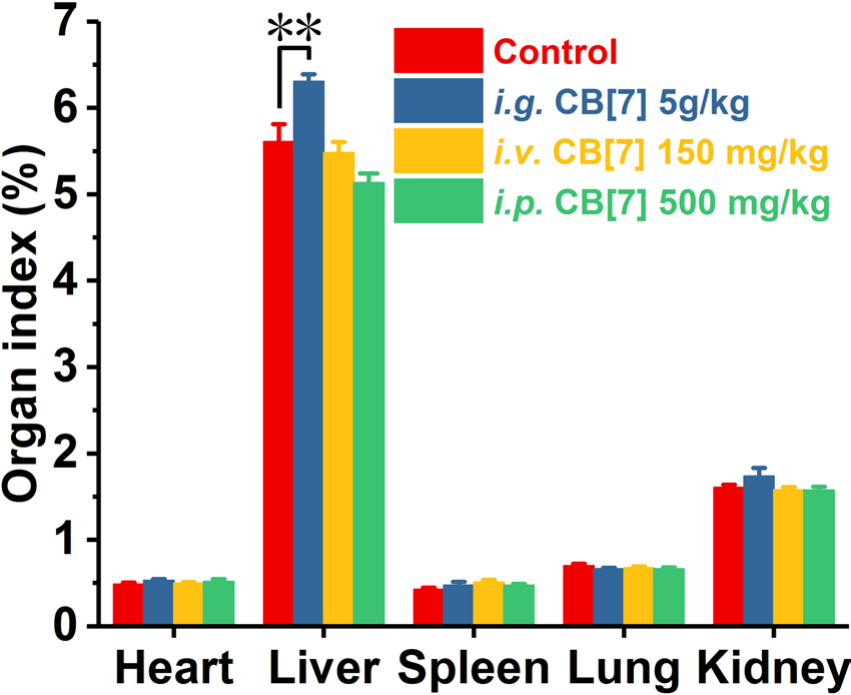
Major organ indices of the mice at 21 days post-administration (*i.g*., *i.v*. or *i.p*.) with CB[7], respectively. **p < 0.01.

**Figure 4:**
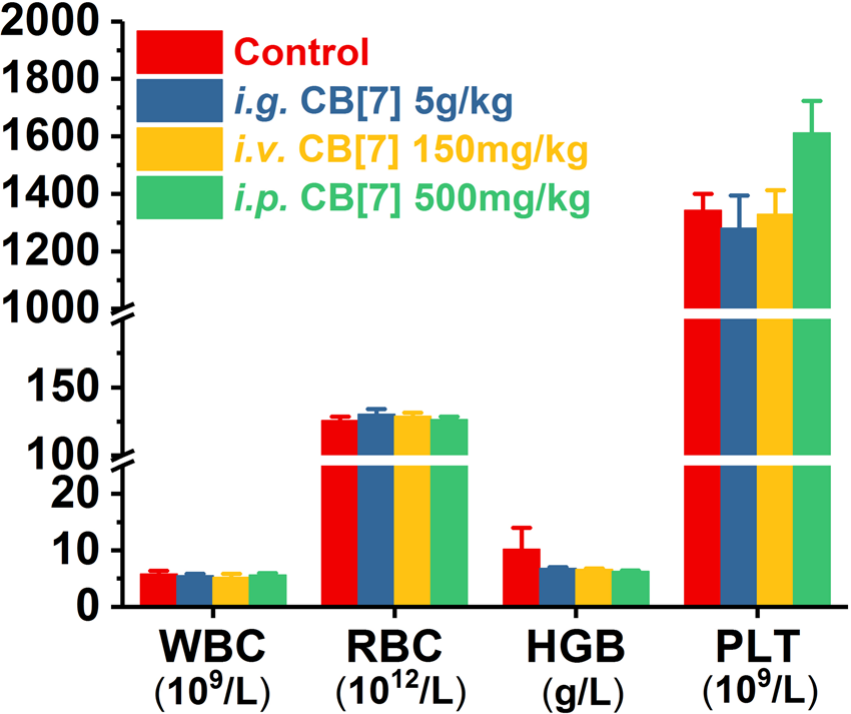
Hematological parameters of the blood samples collected from the mice on Day 21 post *i.g*., *i.v*. and *i.p*. administration with CB[7].

**Figure 5:**
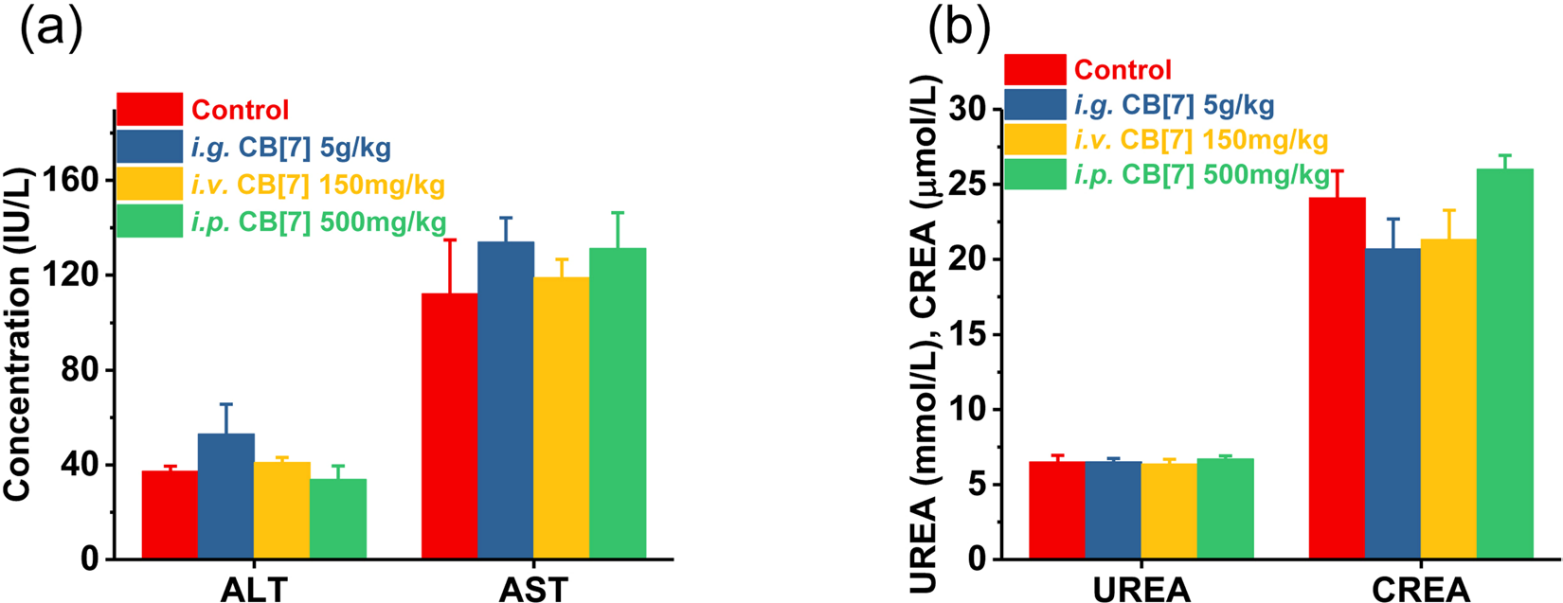
Hepatic (**a**) and renal (**b**) function markers test on the blood samples collected from the mice on Day 21 post *i.g., i.v*. and *i.p*. administration with CB[7], respectively.

**Figure 6:**
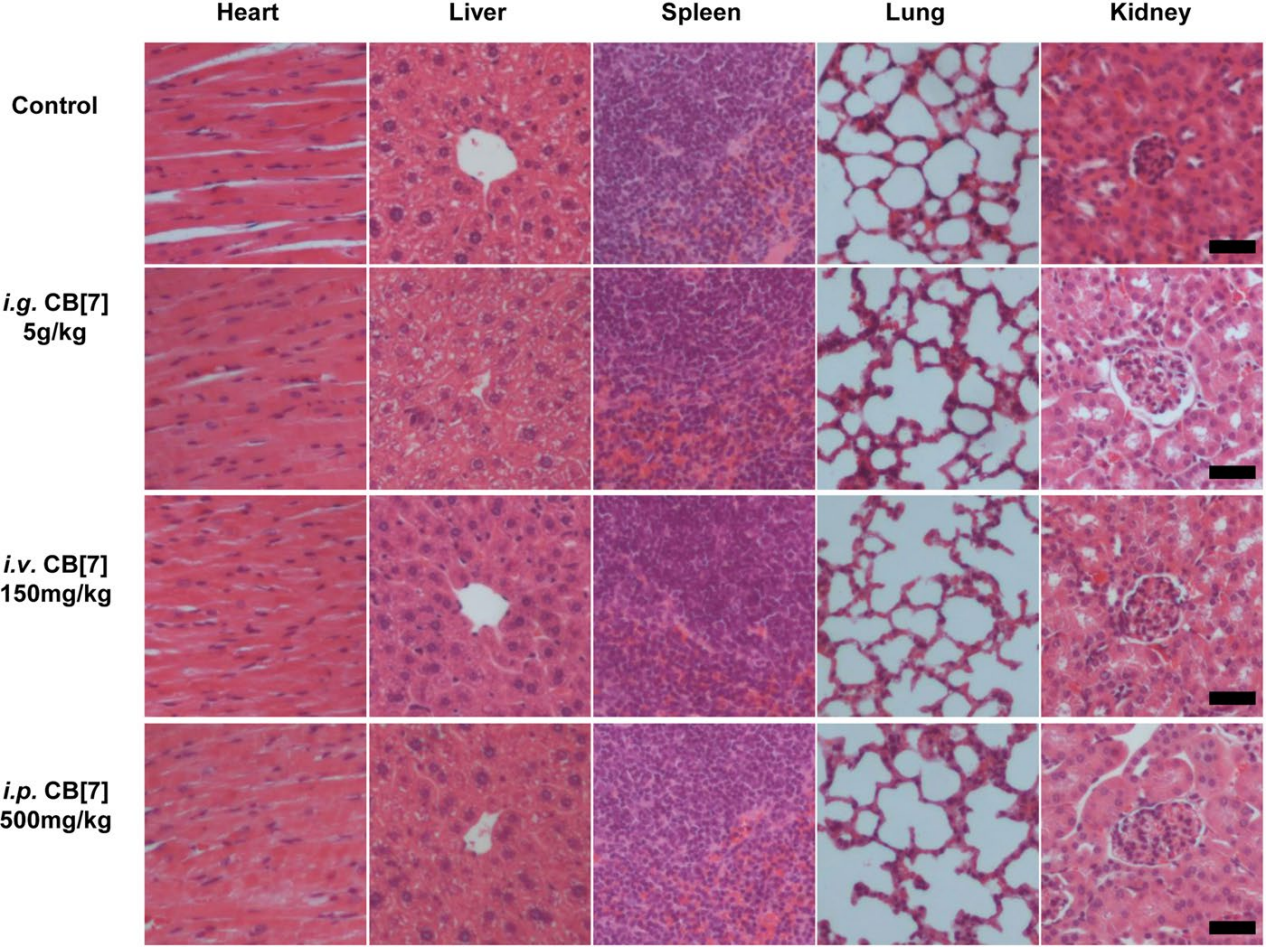
H&E stained sections of major organs from the mice sacrificed on Day 21 post *i.g*., *i.v*. and *i.p*. administration of CB[7], respectively. Scale bar = 100 μm.

In a previous study, mice that were orally administered with a single dose of 600 mg/kg of 1:1 CB[7]/CB[8] mixture did not exhibit any weight loss within 9 days post-administration^30^, implying that there was a good oral biocompatibility profile of CB[7] at a dose of 300 mg/kg. However, this dosage might not be the maximum tolerable oral dose limit, as no dose escalation studies were performed in the previous investigation. Indeed, it is suggested by the Globally Harmonized System (GSH) that a substance would not be labelled as toxic if its median lethal oral dose was >5 g/kg^31^. Herein, to examine whether CB[7] could meet the criteria of being “non-toxic”, we evaluated the acute oral toxicity of CB[7] in mice at the dose of 5 g/kg. Mice were orally administered with a single dose of CB[7] (5 g/kg body weight) or saline (100 μL/10 g, control group). Subsequently, they were weighed every two days to monitor their body-weight changes and behaviours. All mice in this study remained alive within 21 days, and no unusual behaviours or illnesses were observed during the experiment. As shown in Fig. 2, the *i.g*. administration of CB[7] (5 g/kg dose) showed negligible influence on the body weight of the mice, which rose steadily during the 21-day follow-up, comparable to that observed for the control group. Thus, all of the mice were sacrificed after 21 days for additional systemic toxicity evaluation, including haematological, hepatic and renal function biochemical markers tests, as well as for examinations of their organ indices and histological analysis, to examine the potential systemic toxicity of CB[7] at 3-week after acute ingestion. Analysis on the organ indices of the mice sacrificed on Day 21 post CB[7] administration suggested that no significant differences existed between the CB[7]-administered group and control group (Fig. 3), except for a very modest increase (from 5.6% to 6.3%) of the liver index for the mice administered with CB[7] (5 g/kg), which might be an indication of a very minor liver toxicity. Furthermore, haematological counts including white blood cells (WBC), red blood cells (RBC), hemoglobin (HGB) and platelets (PLT) counts from the blood collected from the mice on Day 21 post-administration of CB[7] revealed normal values that were comparable to those in the control group (Fig. 4). In addition, the hepatic and renal function tests for bio-markers including aspartate aminotransferase (AST), alanine aminotransferase (ALT), creatinine (CREA), and urea nitrogen (UREA), of the blood collected from the mice also showed no significant differences in the levels of these biomarkers between the CB[7]-treated group and the control group (Fig. 5a,b). Therefore, *i.g*. administration of CB[7] (at a dose as high as 5 g/kg) did not affect the hepatic and renal functions, in spite of the observation of a very modest increase in the liver index (Fig. 3). In order to further examine the potential influence of CB[7] on various organs, all major organs including the heart, liver, spleen, lungs and kidneys were removed on Day 21 and subjected to histological analysis. As shown in Fig. 6, the hematoxylin and eosin (H&E) stained sections of major organs showed no discernible/detectable injures or signs of inflammatory cells infiltration, similar to those in the control group. Particularly, oral administration of CB[7] did not induce any signs of ulceration in the gastrointestinal (GI) tissues (Figs S1 and S2), thus demonstrating its excellent biocompatibility profile. Taken together, orally administered CB[7] displayed an excellent safety profile in mice, which may further promote investigations of CB[7] in oral drug formulations and delivery. In a previously published article, researchers studied the pharmacokinetics of CB[8] that was administered orally in rats and they found only a very small amount of ^14^C-radiolabelled CB[8] was absorbed into the circulatory system and a large proportion was obtained from the excreted faeces^32^. Therefore, the excellent oral safety profile of CB[7] may also be attributed to its poor absorption in the GI track, as is the case with CB[8].

### Toxicity evaluation of CB[7] with *i.v*. administration

The maximum tolerable dose of CB[7] in mice with a slow *i.v*. administration was reported to be 250 mg/kg from a body-weight change perspective, where the mice experienced a very modest loss of body weight for 4–8 days post-administration, and subsequent recovery^30^. In our study, mice were firstly *i.v*. administered with CB[7]/saline solution at a dose of 300 mg/kg, and severe spasms occurred immediately, resulting in fatality within minutes. Similarly, a single intravenous dose of 200 and 250 mg/kg CB[7] also caused spasms and the mice went into a shock-like state in minutes after administration, consistent with results previously reported^30^. Reducing the dose down to 150 mg/kg did not yield any immediate toxic-effects in mice, and we therefore systematically evaluated the toxicity of CB[7] in mice with an *i.v*. dose of 150 mg/kg. Upon administration with a bolus injection of CB[7]/saline solution, all of the mice remained alive without exhibiting any signs of unusual behaviours or illness during the experiment for 21 days post-administration. As shown in Fig. 2, the body weights of the mice decreased very slightly for the first 3 days post-administration and subsequently rose steadily from Day 5. The organ indices of major organs of the mice sacrificed on Day 21 after *i.v*. injection of CB[7] showed no significant differences in comparison with those of the control group (Fig. 3). Furthermore, analysis of typical hematological parameters as well as hepatic and renal function markers tests of the blood from the mice sacrificed on Day 21 after *i.v*. administration of CB[7] revealed normal values that were comparable to those in the control group (Figs 4 and 5a,b). Moreover, histopathological sections of major organs (including the heart, liver, spleen, lungs and kidneys, as well as the GI track) of the mice showed no discernible/detectable injures and indications of inflammatory cells infiltration, similar to those of the control group (Fig. 6, and Figs S1 and S2). Taken together, these results demonstrated a relatively high *i.v*. biocompatibility of CB[7] (at a dose as high as 150 mg/kg) in mice, as this level of CB[7] *i.v*. injected into the mice did not induce any signs of toxicity through our systemic evaluations.

### Toxicity evaluation of CB[7] with *i.p*. administration

According to a recent report by Day *et al*., upon *i.p*. administration, CB[7] would be rapidly absorbed into blood circulatory system and distributed into all of the major organs, and very quickly excreted via the kidneys^32^. With this *i.p*. pharmacokinetic profile of CB[7] in place, it is equally important to understand the toxicity profile of CB[7] *i.p*. administered in mice. In our study, mice were first *i.p*. injected with CB[7]/saline solution at the dose of 750 mg/kg, and the mice exhibited severe spasms within minutes, and were followed by death. The mice behaved and died in a similar manner as the mice *i.v*. injected with a high dose of CB[7] (300 mg/kg). Subsequently, the dose was lowered down to 500 mg/kg and no spasms or fatalities were observed. After *i.p*. injection of CB[7] at this dose (500 mg/kg), all of the mice remained alive without exhibiting any signs of illness or unusual behaviors during the experiment for 21 days post-administration. As shown in Fig. 2, the average body weight of the mice decreased very modestly on Day 1 and quickly rose back to normal again from Day 3 to the end of the experiment, Day 21. The mice were sacrificed on Day 21, and organ indices of the major organs of these mice were analyzed. The major organ indices of the mice injected with CB[7] were comparable with those of the control group, as shown by Fig. 3. The hematological parameters (blood cell counts), and hepatic and renal function biomarkers tests of the blood collected from the mice on Day 21 afforded normal values for the *i.p*. CB[7] treated group, which were comparable to those in the control group (Figs 4 and 5a,b). Moreover, histopathologic sections of major organs (including the heart, liver, spleen, lungs and kidneys, as well as the GI track) of the mice *i.p*. injected with CB[7] showed no discernible/detectable injures or signs of inflammatory cells infiltration, similar to those in the control group (Fig. 6, and Figs S1 and S2). These results indicated that a single dose of as high as 500 mg/kg CB[7] with *i.p*. administration exhibited negligible toxicity in mice, suggesting an excellent *i.p*. biocompatibility profile *in vivo*.

## Discussion

This study represents the first systematic evaluation on the safety and biocompatibility of CB[7] in mice via *i.g*., *i.v*. and *i.p*. administration, respectively. We have demonstrated that CB[7] *i.g*., *i.v*. and *i.p*. administered with the dose as high as 5 g/kg, 150 mg/kg and 500 mg/kg, respectively, exhibited excellent safety profiles in mice. This finding was reached through examinations of the body-weight changes for 21 days after dose-administration for acute toxicity evaluation, and organ indices, hematological parameters, hepatic and renal functions, as well as major organ histology of the mice on Day 21 after administration with CB[7] for 3-week toxicity evaluation. Such an excellent safety profile of CB[7] with *i.g*., *i.v*. and *i.p*. administration may provide important foundations for further investigations and even clinical applications of CB[7] in the biomedical field.

## Methods

### Animals

All studies were conducted with the approval by the Animal Ethics Committee at the Third Military Medical University, according to the Animal Management Rules of the Ministry of Health of the People’s Republic of China (No. 55, 2001) and the guidelines for the Care and Use of Laboratory Animals of the Third Military Medical University. Male Balb/c mice were obtained from the Animal Centre of the Third Military Medical University and housed at a 12-hour light/12-hour dark cycle. Animals were randomly divided into groups based on their body weight and received diet and water ad libitum.

### Synthesis of CB[7]

CB[7] was synthesized and separated via the previously reported methods^9,10,33^.

### Acute toxicity evaluation of CB[7] with* i.g.*, *i.v.* and *i.p.* administration

Male Balb/c mice (8–10 weeks old) with body weights of 20–25 g were randomly separated into four groups (*n* = 6 for each group) and fasted for 8 h prior to the experiments. A 500 mg/mL CB[7]/saline suspension was prepared, and mice were orally administered with a 100 μL suspension/10 g body weight (5 g/kg) CB[7] for the *i.g*. CB[7] group. In the *i.p*. CB[7] group, 50 mg/mL CB[7]/saline solutions were prepared and passed through a 0.22 μm filter membrane and the mice were peritoneally injected with 100 μL solution/10 g body weight. In the *i.v*. CB[7] group, 30 mg/mL CB[7]/saline solutions were prepared and passed through a 0.22 μm filter membrane and mice were intravenously administered with 50 μL solution/10 g body weight. For the control group, the mice were only administered with the same amount of saline that was used in the treatment group (*n* = 6). Mice were weighed every two days, and their behaviours were observed for any signs of illness each day. In order to conduct a more systemic evaluation on potential toxicity of CB[7] at 3-week after CB[7] ingestion, animals were sacrificed after 21 days. Blood samples were collected for the hematological analysis. In addition, serum was separated for the hepatic and renal functional marker tests. Major organs including the heart, liver, kidneys, lungs and spleen were collected and weighed for the organ index calculation. GI tissues were also harvested and washed with phosphate buffer saline (PBS). Duodenum, jejunum and ileum with a length of 5 cm for each section were separated. Sections of major organs and GI tissues were prepared and stained with hematoxylin-eosin (H&E) for histopathological analysis.

### Calculation of the organ index

$${\rm{Organ}}\,{\rm{index}}=\frac{{\rm{weight}}\,{\rm{of}}\,{\rm{the}}\,{\rm{organ}}}{{\rm{body}}\,{\rm{weight}}}\times 100 \% $$

### Determination of the hematology parameters

Blood samples were collected into EDTA spray-coated tubes, WBC, RBC, PLT and HGB were analyzed with an automated hematology analyzer (Sysmex KX-21, Sysmex Co., Japan).

### Biochemical markers of liver and kidney function

Blood samples were collected into covered test tubes and clotted by leaving them undisturbed for 30 min to 1 h at room temperature. Serum samples were separated and collected by centrifuging the clotted blood samples at 2000 *g* for 10 min. The serum concentrations of the routine hepatic and renal function markers including AST, ALT, CREA, and UREA were subsequently quantified (Roche Cobas C501, Roche Co., Switzerland).

### Histopathology

The major organs and GI tissues of the mice were fixed in 10 wt% neutral formalin for 24 h, embedded in paraffin and cut into 6-μm sections. The slides were stained with H&E and examined with an optical microscope (Nikon Digital Sight, paired with NIS-Elements BR software).

### Statistical analysis

Data are presented as means ± SEM. Two-tailed, unpaired Student’s *t* tests were performed for the comparisons between two sample sets with PASW Statistics 18.0.

### Data availability Statement

All data generated or analysed during this study are included in this published article and Supplementary Information.

## Electronic supplementary material

Supplementary Information
