# Supplementary material for: A systematic evaluation of the biocompatibility of cucurbit[7]uril in mice
Source: Sci Rep. 2018 Jun 11;8:8819. doi: 10.1038/s41598-018-27206-6 (PMC5995857; doi:10.1038/s41598-018-27206-6)
Supplement: Supplementary file 1 — Supplementary Information [file 41598_2018_27206_MOESM1_ESM.pdf]

# Supporting Information

# A systematic evaluation of the biocompatibility of cucurbit[7]uril in mice

**Xiangjun Zhang[1], Xiaoqiu Xu[2,3], Shengke Li[1], Lian-Hui Wang,[4] Jianxiang Zhang[2,3,*], and Ruibing Wang[1,*]**

[1]State Key Laboratory of Quality Research in Chinese Medicine, and Institute of Chinese Medical Sciences, University of Macau, Taipa, Macau, China
[2]Department of Pharmaceutics, College of Pharmacy, Third Military Medical University, Chongqing 400038, China
[3]Institute of Materia Medica, College of Pharmacy, Third Military Medical University, Chongqing 400038, China
[4]Key Laboratory for Organic Electronics and Information Displays, Institute of Advanced Materials, Nanjing University of Posts & Telecommunications, Nanjing 210046, China.

[*]Corresponding authors: rwang@umac.mo or jxzhang@tmmu.edu.cn

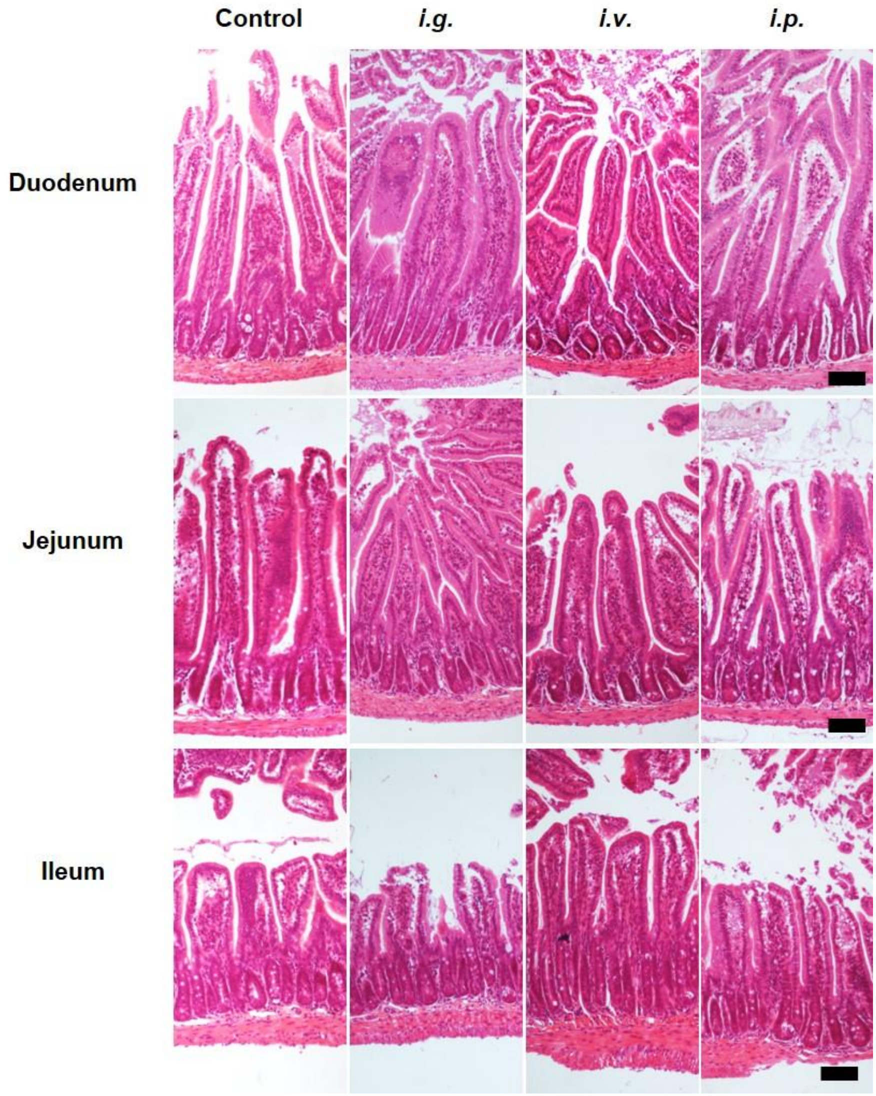


**Fig. S1** H&E stained sections of intestine tissues collected from mice 21 days after *i.g.*, *i.v.* and *i.p.* administration with CB[7]. Scale bar = 200 μm.

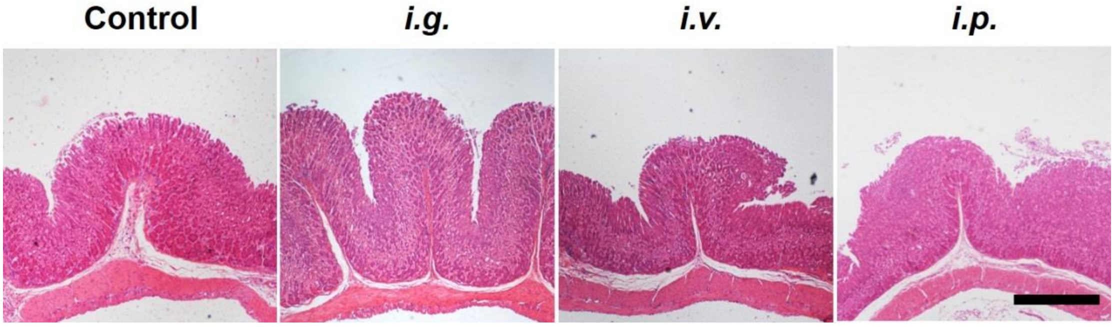


**Fig. S2** H&E stained sections of stomach from mice 21 days after *i.g.*, *i.v.* and *i.p.* administration with CB[7]. Scale bar = 500 μm.
